# Caspase-7 and acid sphingomyelinase: partner repairmans of gasdermin and perforin pores

**DOI:** 10.1038/s41392-022-01144-2

**Published:** 2022-08-22

**Authors:** Ke Jin, Guofang Shen

**Affiliations:** 1 Laboratory of Human Diseases and Immunotherapies, West China Hospital, Sichuan University, 610041 Chengdu, China; 2 Institute of Immunology and Inflammation, Frontiers Science Center for Disease-related Molecular Network, West China Hospital, Sichuan University, 610041 Chengdu, China; 3 Department of Hematologic Malignancies Translational Sciences, Beckman Research Institute of City of Hope, Duarte, CA 91010 USA

**Keywords:** Inflammation, Infection

**Fig. 1:**
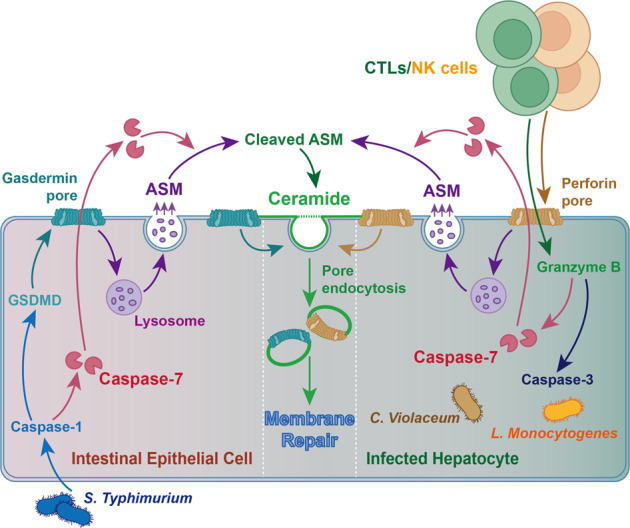
Caspase-7 antagonizes gasdermin D and perforin pores in inflammatory and apoptotic models and maintains cell membrane integrity by cleaving and activating acid sphingomyelinase (ASM) to produce sufficient ceramide for plasma membrane repair. Caspase-7 can be activated by pyroptotic caspase-1 in intestinal epithelial cells (IECs) (left), or acts downstream of granzyme B in infected hepatocytes which suffer from perforin-pore-mediated attack by natural killer (NK) cells or cytotoxic T lymphocytes (CTLs) (right). Then caspase-7 cleaves ASM, which generates more ceramide for endocytic repair of gasdermin D and perforin pores to facilitate IEC extrusion or target cell (infected hepatocytes in this model) apoptosis. *C*. *Violaceum*, *Chromobacterium Violaceum*; *L*. *Monocytogenes*, *Listeria Monocytogenes*

Recently, a work published in Nature by Kengo Nozaki et al. revealed a novel mechanism for plasma membrane pores’ repair mediated by caspase-7.^1^ In this work, they found caspase-7 facilitated Intestinal epithelial cell (IEC) extrusion and repaired gasdermin D (GSDMD) and perforin pores via activating acid sphingomyelinase (ASM). The pores repair mechanism mediated by caspase-7 is independent of caspase-3 activity (Fig. 1).^1^

IEC extrusion plays a critical role in maintaining intestinal barrier integrity, homeostatic cell turnover, and defense against pathogens. Conventionally, caspase-3 and caspase-7 are involved in the execution step of apoptosis.^2^ Caspase-3 is thought to serve as the mighty executioner of apoptosis, while caspase-7 is typically considered as a backup for caspase-3.^1^ The differences in the biological functions that caspase-3 and -7 mediate are largely unknown. Currently, we only know that caspase-3 and caspase-7 show differential activities towards some substrates, including X-linked inhibitor of apoptosis (XIAP), gelsolin, BH3-interacting domain death agonist (BID), p23 and caspase-6.

Kengo Nozaki et al. confirmed that caspase-7 was highly expressed in IECs, and many cleaved caspase-7 positive IECs accumulated in caecum after *Salmonella enterica* subsp. *enterica serovar* Typhimurium (*S*. *Typhimurium*) infection in mice. Activated caspase-7 is dominantly expressed in extruding IECs when mice were exposed to *S*. *Typhimurium*, suggesting that IEC extrusion may require the involvement of caspase-7. IEC extrusion initiated by *S*. *Typhimurium* infection and caspase-1 activation can be mimicked by IEC organoid stimulated with an engineered toxin FlaTox. Therefore, the authors utilized organoid model to study IEC extrusion in their subsequent study. With FlaTox treatment in IEC organoids, they found caspase-7 was cleaved quickly. This process was delayed in *Casp1*^−/−^*Casp11*^−/−^ organoids but was not affected in *gasdermin d*^−/−^ (*Gsdmd*^−/−^) or *Casp3*^−/−^ organoids, indicating that caspase-7 was activated by caspase-1 instead of caspase-3.

Once activated by canonical inflammasomes, caspase-1 cleaves GSDMD to form pores on the plasma membrane. Pores formed by GSDMD will trigger pyroptosis, one way of programmed cell death, which is different from canonical apoptosis in cell death.^3^ In this study, the authors answered following questions. Whether GSDMD pore repair was related to caspase-7? They first detected the pore permeability assayed by propidium iodide (PI) uptake experiment. They found that PI uptake was significantly increased in FlaTox-treated *Casp7*^−^^/−^ organoids. In addition, GSDMD and caspase-7 were cleaved quickly after FlaTox treatment in the IEC organoid. Therefore, they concluded that caspase-7 would impede the function of caspase-1 mediated formation of gasdermin pores. How did caspase-7 interfere with pore-forming processes? Caspase-7 has been proved to cleave ASM and enhance its sphingomyelin-catalyzing activity. ASM-driven lysosome endocytosis can repair the plasma membrane. Thus, the authors linked caspase-7 to ASM and hypothesized that GSDMD pore repair required caspase-7 and ASM activation. The authors proved that ASM was indeed cleaved by caspase-7, and that the cleavage site was D249 in IEC organoids. As shown in their study, there was no ASM cleavage in *Smpd1*^*D249A/D249A*^ organoids after treatment of FlaTox. Was ASM directly involved in membrane pore repair? Sphingomyelin is one of the important components of the cell membrane, and ceramide, sphingosine, and sphingosine-1-phosphate are sphingomyelin-derived metabolites. ASM participates in converting sphingomyelin into ceramide, which mediates plasma membrane endocytosis. Therefore, the plasma membrane pores repair may be mediated by ASM via ceramide production. Consistently, the authors found that ceramide was strongly expressed in extruding IECs after *S*. *Typhimurium* infection, while no ceramide was enriched in extruding IECs in *casp7*^−^^*/−*^ mice. They also found the treatment of ceramide would rescue the abnormal phenotypes of *casp7*^−^^*/−*^ organoids, such as normalizing their extrusion initiation time, PI staining intensity, and rupture percentage. These results demonstrated that caspase-7 repaired GSDMD pores via ceramide produced by activated ASM.

Another interesting question raised by the authors was how caspase-7 interacted with ASM and subsequently cleaved ASM? Caspase-7 resides in the cytosol, whereas ASM resides in the lysosomal lumen. If caspase-7 can interact with ASM successfully, it needs to cross the membrane, and the specific mechanism remains unclear. The authors hypothesized that GSDMD might be a ‘channel’ that linked caspase-7 to ASM. Consistent with their hypothesis, ASM could not be cleaved in *Gsdmd*^−/−^ organoids by treatment of FlaTox, and ceramide production was also lost in *Gsdmd*^−/−^ mice after *S*. *Typhimurium* infection. These results supported their hypothesis that caspase-7 could cross the GSDMD pore to interact with and cleave ASM, and that GSDMD was a prerequisite for this process. They further proved caspase-7 could extenuate intestinal pathology such as mitigating intestinal damage induced by *S*. *Typhimurium* and reducing colitis induced by dextran sodium sulfate (DSS) in mice models. It is worth noting that caspase-7 could reduce intestinal damage, but not lower the *S*. *Typhimurium* burden. It means that caspase-7 mitigates intestinal pathology by repairing gasdermin pores instead of eliminating pathogens.

Perforin, a cell toxin released by cytotoxic T lymphocytes (CTLs) and natural killer (NK) cells, mediates pore-forming on the plasma membrane. Perforin pore can initiate granzymes-induced apoptosis.^4^ To confirm whether ASM activation is the core evolved function of caspase-7, the authors studied this pore-forming process mediated by perforin. As expected, they found caspase-7 could activate ASM after NK cell and CTL perforin attack. Meanwhile, caspase-7 also promoted pathogen clearance by NK cells and CTLs which is different from the case of gasdermin pore repair. The authors noted that caspase-7 and perforin appeared earlier than GSDMD. Therefore, the authors speculated that the original evolutionary function of caspase-7 was to counteract the plasma membrane pore caused by perforin, while the secondary role of caspase-7 was to offset the GSDMD pore which appeared later in the evolution. This conjecture may explain why caspase-7 is not only involved in perforin pore repair, but also involved in perforin-mediated pathogen clearance.

Another related work by Farzaneh Ghazavia et al. recently reported that caspase-3 and caspase-7 are dispensable for IEC turnover and homeostasis at a steady state.^5^ Considering the results from Kengo Nozaki et al. together, we can conclude that caspase-7 exerts the function of pore repair in pathological condition of IECs, but is redundant in the physiological condition of IECs. When working as an executioner of apoptosis, the pore repair function of caspase-7 seems to be shut down by some unknown mechanism. Overall, this study illustrates a novel role of caspase-7 in IECs. Caspase-7 is not only an executioner of apoptosis, but also a repairman of gasdermin and perforin pores that helps cells to maintain an intact membrane for a certain period.
